# Eurythmy therapy increases specific oscillations of heart rate variability

**DOI:** 10.1186/s12906-015-0684-6

**Published:** 2015-06-06

**Authors:** Friedrich Edelhäuser, Antje Minnerop, Barbara Trapp, Arndt Büssing, Dirk Cysarz

**Affiliations:** Integrated Curriculum for Anthroposophic Medicine, University of Witten/Herdecke, Gerhard-Kienle-Weg 4, 58313 Herdecke, Germany; Chair for Theory of Medicine, Integrative and Anthroposophic Medicine, University of Witten/Herdecke, Herdecke, Germany; Institute of Integrative Medicine, University of Witten/Herdecke, Herdecke, Germany

**Keywords:** Heart rate variability, Cardiac autonomic regulation, Mind-body therapies, Exercise, Eurythmy therapy, Anthroposophic medicine

## Abstract

**Background:**

Mind-body therapies are beneficial for several diseases (*e.g.* chronic pain, arterial hypertension, mood disorders). Eurythmy therapy (EYT) is a mind-body therapy from Anthroposophic Medicine. In each EYT exercise a short sequence of body movements and simultaneous guided imagery is repeated several times. In this study, the simultaneous effects of two different EYT exercises on cardiac autonomic regulation as assessed by spectral analysis of heart rate variability (HRV) were investigated.

**Methods:**

Twenty healthy subjects (age: 29.1 ± 9.3 years, 13 female) performed two different EYT exercises (EYT-A and EYT-B) for 8 min. Each EYT exercise was compared against two matched control exercises: control exercise 1 (CE1-A and CE1-B) consisted of a repetition of the body movements of the EYT exercise but without guided imagery, control exercise 2 consisted of walking on the spot (CE2-A and CE2-B). Spectral power of HRV during each exercise was quantified on the basis of Holter ECG recordings.

**Results:**

During EYT-A the frequency of the peak oscillation in the very low frequency (VLF) band matched the repetition rate of the sequence of body movements (0.02 Hz). Low frequency (LF) oscillations were augmented when compared to the control exercises (EYT-A: 7.31 ± 0.84, CE1-A: 6.98 ± 0.90, CE2-A: 6.52 ± 0.87 ln ms^2^, p < 0.05). They showed a peak frequency at 0.08 Hz indicating that the body postures had an impact in HRV. Performing EYT-B increased VLF oscillations when compared to the control exercises (EYT-B: 9.32 ± 0.82, CE1-B: 6.31 ± 0.75, CE2-B: 6.04 ± 0.80 ln ms^2^, p < 0.05). The frequency of the peak oscillation again matched the repetition rate of the sequence of body movements (0.028 Hz).

**Conclusions:**

The repetition of the sequence of body movements of both EYT exercises clearly affected cardiac autonomic regulation in a rhythmic manner according to the stimulus of the specific body movements of each EYT exercise. These results offer a physiological basis to develop a rationale for specific clinical indications of these EYT exercises such as stress reduction or prevention of hypertension.

**Trial registration:**

Clinical trials registration number: DRKS00006760 (registered on 10/10/2014, *i.e.* retrospective registration); view details at http://www.drks.de/DRKS00006760

## Background

Mind-body treatments such as mindfulness meditation and yoga have been intensively investigated during recent years because they are often associated with psychological stabilization and lower level of stress [1–4]. Specific mind-body therapies such as Tai Chi and Eurythmy therapy (EYT) comprise relatively slow body movements in conjunction with aspects of meditation in terms of accompanying guided imagery. The body movements play an essential role in these therapies. Usually body movements are based on conscious and subconscious perception of the environment and on subconscious perception of motion [5]. In these therapies the perception-based conception of movements is brought into attention through mental imagery of the movements [6]. It has been shown that the intensity and kinaesthetic modality of mental imagery improves movement performance [7]. Furthermore, normal body movements as well as mental imagery of body movements activate similar areas of the brain indicating a close relationship between active movements and imagery of movements [8]. Thus, imagery of movements and active movements can also be arranged for therapeutic purposes and mind-body therapies like Tai Chi and EYT seem to facilitate such arrangements. Here, we will focus on EYT as an active intervention.

Eurythmy therapy (Eu-rhythmy means harmonious rhythm) originates in Western culture and was developed in the first decades of the 20th century as a part of Anthroposophic Medicine. The specific EYT movements are based on gestures of the human movement spectrum which are intensified by focussing on specific aspects of the movements. Accompanying guided imagery is tightly connected to the movements to incur feelings and thoughts which are appropriate for the particular sequence of movements. In practice, EYT consists of a relatively short sequence of body movements with accompanying guided imagery. This sequence is continuously repeated several times during the therapy. Evidence is growing that EYT, among others, is useful for the reduction of symptoms in patients with various chronic diseases [9]. Moderately stressed adults showed an improvement of their quality of life after a six-weeks EYT intervention [10]. Also, children with attention deficit hyperactivity disorder may benefit from EYT [11]. A recent systematic review stated that EYT may be deemed a relevant add-on in a therapeutic concept [12].

The evidence with respect to effects of EYT on cardiac autonomic regulation and cardiovascular health is sparse. In healthy subjects very low frequency (VLF) and low frequency (LF) oscillations of heart rate variability (HRV) increased during the performance of EYT exercises compared to conventional ergometer training [13]. After healthy subjects completed a 6-week EYT training (a total of 10 h of EYT), the absolute values of LF and high frequency (HF) oscillations decreased when compared to baseline before the training whereas the LF and HF power relative to the total power increased [14]. Furthermore, during night time after EYT training the HF and LF power relative to LF + HF power decreased when compared to night time values before the training [15]. These results seem to support effects of EYT on cardiac autonomic regulation after the EYT training.

The studies on EYT to date have not unambiguously addressed the impact of body movements during the performance of different EYT exercises on cardiac autonomic control (simultaneous effects). Specifically, it is not clear whether the repetition of the sequence of body movements, *i.e.* a repetitive (or rhythmic) stimulus, has a repetitive (rhythmic) impact on cardiac autonomic control. Furthermore, different body postures during one sequence of body movements may also lead to different effects on cardiac autonomic control. Hence, in this exploratory study we focus on simultaneous effects of two different EYT exercises on cardiac autonomic regulation as assessed by the analysis of HRV. One EYT exercise consists of a sequence of different body postures which are subsequently taken whereas the other EYT exercise consists of a sequence of continuous body movements. Both sequences are repeated several times during the exercise. We hypothesize that both EYT exercises show different effects on cardiac autonomic control primarily imposed by the repetition of the specific sequence of body movements during each EYT exercise.

## Methods

### Subjects

Twenty healthy subjects aged 20 to 51 years were enrolled in the study (mean: 29.1 ± 9.3 years; 13 female, 7 male). None of the subjects had any history of cardiovascular diseases, in particular no hypo- or hypertension or antiarrhythmic therapy. All subjects were inexperienced with respect to EYT exercises. They abstained from caffeine and nicotine during the day of the study. All subjects gave written informed consent. The study was approved by the ethics committee of the University of Witten/Herdecke (registration nr. 22/2007).

### Procedure

The EYT exercises were investigated using the following experimental procedure:

Rest – **EYT** – Rest – **CE1** – Rest – **CE2** – Rest.

Two different EYT exercises (EYT-A and EYT-B, see below) were investigated and, hence, each subject carried out the procedure twice. CE1 and CE2 denote control exercises: during control exercise 1 (CE1) the same sequence of movements as during the EYT exercise was repeated several times but without guided imagery. Hence, CE1 was a control exercise closely adapted to the respective EYT exercise (further denoted as CE1-A and CE1-B). Control exercise 2 (CE2) consisted of walking on the spot as a non-specific control exercise (*i.e.* no repetition of a specific sequence of body movements; they are further denoted as CE2-A and CE2-B). All exercises could be performed by the subjects with normal efforts. EYT exercises and CE1 were demonstrated by an experienced therapist whereas CE2 was carried out without guidance. All exercises (EYT exercises, CE1 and CE2) had to be performed with similar effort. Rest denotes sitting on a chair. Each part of the procedure lasted 8 min and, hence, the total duration of the procedure was approximately 60 min. The subjects were asked not to speak throughout the procedure but there were no instructions with respect to breathing modalities.

The procedure was performed by four subjects simultaneously for each EYT exercise to maintain proper guidance of the exercises by the therapist. Hence, the procedure had to be repeated five times to include all subjects for a single EYT exercise. A video was recorded of each procedure to aid in the retrospective analysis of the number of sequences of the body movements during each exercise.

### Eurythmy therapy (EYT)

Each EYT exercise consists of a repetition of a specific sequence of body movements in conjunction with appropriate guided imagery. We examined two different EYT exercises termed *‘I Think The Saying’* (EYT-A) and *‘Migraine B’* (EYT-B) which are commonly used by eurythmy therapists. They differ with respect to the body movements and the frequency of repetitions. A certified and experienced eurythmy therapist demonstrated the sequence of movements of each EYT exercise and the subjects performed these movements simultaneously with the therapist. At the same time the therapist also conducted the guided imagery that was appropriate to the movements. The therapist repeated the sequence of movements (and the guided imagery) several times during the 8 min.

**Fig. 1:**
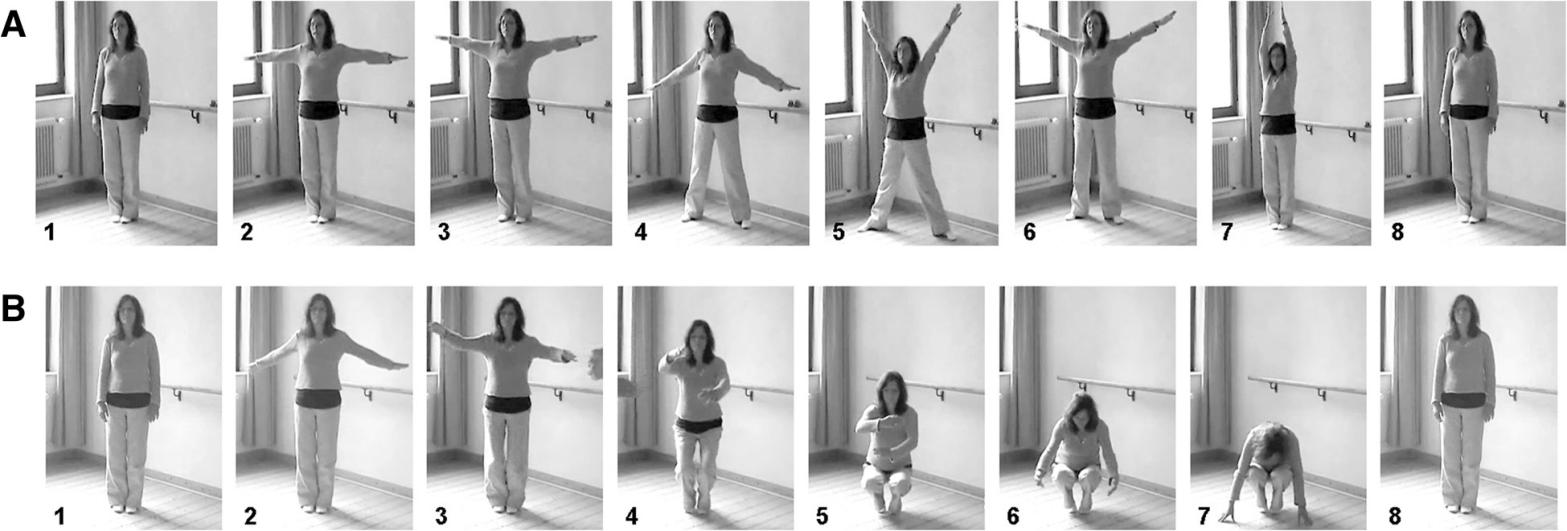
Sequence of movements of the EYT exercises **a** EYT-A (*‘I Think The Saying’*) and **b** EYT-B (*‘Migraine B’*) which is repeated several times during the exercise. Note that during *‘I Think The Saying’* different body postures are successively taken (pictures 2 to 7) whereas during *‘Migraine B’* the movement is smooth with the different postures shown in pictures 2 to 7. For further information see the text. Sequence of photos reproduced by courtesy of author BT

The exercises started with an initial posture (see Fig. 1A, picture 1: standing upright in a quiet and calm manner). During the *‘I Think The Saying’* exercise, a movement sequence consisted of 6 different postures which were subsequently taken. Each posture (pictures 2 to 7) was held for some seconds and was accompanied by a specific spoken phrase to guide the inner activity. Then the next posture was taken. The sequence was finished by returning to the initial posture (picture 8). This sequence was repeated several times during the 8 min. The exercise *‘Migraine B’* consisted of a continuous slow body movement. A movement sequence started with an upright position in a quiet and calm manner (see Fig. 1B, picture 1). The body was continuously moved as shown in pictures 2 to 7. An essential part of the movement consisted of slowly squatting and returning to upright posture again. At the same time the therapist guided the inner activity such that feelings of ‘warmth’ and imagination of ‘light’ arise between the arms in front of the heart (picture 5), and are subsequently ‘released’ down to the floor. The movement sequence ended by taking the initial posture. Again, the sequence of body movements (and the guided imagery) was repeated several times during the 8 min.

### Heart rate variability (HRV)

An electrocardiogram (ECG) was recorded throughout the procedure at a sampling rate of 4096 Hz using a Holter device (MK3 Holter recorder, Schiller Engineering, Austria). The Holter device had an automatic R-peak detection. The ECG and the times of the R-peaks were transferred to a PC for further analysis. The times of the automatically identified R-peaks were visually checked and corrected if necessary (<0.1 % of all identified R-peaks). EYT, CE1, CE2 and Rest were analyzed as follows: the first two minutes of each part were omitted to minimize effects caused by adaptations and the subsequent 5 min were analyzed.

Spectral analysis of the RR-interval series was deemed appropriate for the analysis because the EYT exercises consist of repetitions of a specific sequence of body movement and these repetitions were deemed to have a repetitive, *i.e.* rhythmic, impact on the RR-interval series during the exercise. The power spectral analysis was carried out as follows. The series of normal-to-normal beat times (RR-intervals) served as the basis for further calculations. The RR-interval series was transformed into a time series (sampling rate: 4 Hz) and subsequently a Fourier transformation was applied. The frequency components were calculated according to the standard definitions of the guidelines [16] to enable comparability with other studies. The following frequency ranges were used: very low frequency (VLF: <0.04 Hz), low frequency (LF: 0.04-0.15 Hz) and high frequency oscillations (HF: 0.15-0.4 Hz). Furthermore, the frequency of the peak oscillations was calculated for each frequency band to investigate the impact of the repetitions of the sequence of body movements during each EYT exercise on cardiac autonomic control. In addition, HRV was quantified by the average RR-interval and the standard deviation of the RR-intervals (SDNN) as a measure of overall variability. All calculations were carried out using Matlab (The Mathworks, Natick, Mass., USA).

### Statistics

In this study, all values are expressed as mean ± standard deviation. The frequency domain parameters (VLF, LF, HF) were transformed by taking the natural logarithms because their distributions were skewed. The following conditions were compared: HRV during EYT, CE1, CE2, and Rest at the beginning of the procedure. Changes between these conditions were quantified by a non-parametric analysis of variance (Friedman-test). In case of significant changes, the pair wise comparisons were calculated taking the Bonferroni-correction into account. A p < 0.05 was considered statistically significant.

## Results

All subjects were able to perform the EYT exercises (and the accompanying control exercises) as demonstrated by the therapist.

### EYT-A exercise (“I think the saying”)

Reviewing the recorded videos showed that the sequence of body movements during the EYT-A exercise was repeated 10 times on average during the 8 min (*i.e.* approx. 48 s per cycle corresponding to approx. 0.02 Hz; note that the averaging was carried out with respect to the different subject groups) whereas during CE1-A the sequence was repeated 19 times on average (*i.e.* approx. 25 s per cycle corresponding to approx. 0.04 Hz). During EYT-A, CE1-A and CE2-A the average RR-interval was similar (739 ms, see Table 1), *i.e.* the work load was equal during these exercises. The longest RR-interval appeared during Rest-A (847 ms).

**Table 1:** Measures of heart rate variability. All values are mean ± SD

	*EYT-A: ‘I Think The Saying’*			
	EYT-A	CE1-A	CE2-A	Rest-A
RR [ms]***	739 ± 82	731 ± 73	744 ± 97	847 ± 109^a,b,c^
SDNN [ms]***	61 ± 25	54 ± 22	44 ± 16^a,d^	67 ± 36
VLF [ln ms^2^]	6.68 ± 0.67	6.62 ± 0.80	6.42 ± 0.67	6.92 ± 0.75
LF [ln ms^2^]***	7.31 ± 0.84^b,c,d^	6.98 ± 0.90	6.52 ± 0.87	6.94 ± 1.09
HF [ln ms^2^]**	5.51 ± 1.00	5.44 ± 0.90	5.04 ± 0.88	6.17 ± 1.08^b,c^
Peak osc. VLF [Hz]**	0.019 ± 0.008	0.028 ± 0.013^c,d^	0.013 ± 0.008	0.016 ± 0.011
Peak osc. LF [Hz]	0.081 ± 0.020	0.078 ± 0.027	0.079 ± 0.020	0.090 ± 0.025
Peak osc. HF [Hz]*	0.178 ± 0.037^c^	0.208 ± 0.063	0.238 ± 0.081	0.226 ± 0.062
	*EYT-B: ‘Migraine B’*			
	EYT-B	CE1-B	CE2-B	Rest-B
RR [ms]***	774 ± 100^c^	730 ± 96	620 ± 117	878 ± 109^b,c^
SDNN [ms]***	138 ± 43	118 ± 46	33 ± 14^a,b,d^	73 ± 36^a^
VLF [ln ms^2^]***	9.32 ± 0.82^b,c,d^	6.31 ± 0.75	6.04 ± 0.80	7.05 ± 1.39
LF [ln ms^2^]***	7.91 ± 1.00	8.98 ± 1.12^d^	5.47 ± 1.29^a,b^	6.97 ± 1.07
HF [ln ms^2^]***	6.68 ± 1.04	6.56 ± 1.05	3.82 ± 1.39^a,b,d^	6.39 ± 1.07
Peak osc. VLF [Hz]***	0.026 ± 0.004^c,d^	0.018 ± 0.013	0.008 ± 0.007	0.012 ± 0.009
Peak osc. LF [Hz]**	0.058 ± 0.021^d^	0.070 ± 0.011	0.072 ± 0.025	0.090 ± 0.030
Peak osc. HF [Hz]**	0.185 ± 0.048	0.185 ± 0.041	0.206 ± 0.075	0.236 ± 0.058^a,c^

‘Peak osc.’ denotes the peak oscillation in the respective frequency band

* p_Friedman_ < 0.05, ** p_Friedman_ < 0.01, *** p_Friedman_ < 0.001

^a^ *p* < 0.05 *vs.* EYT, ^b^ *p* < 0.05 *vs.* CE1, ^c^ *p* < 0.05 *vs.* CE2, ^d^ *p* < 0.05 *vs.* Rest

**Fig. 2:**
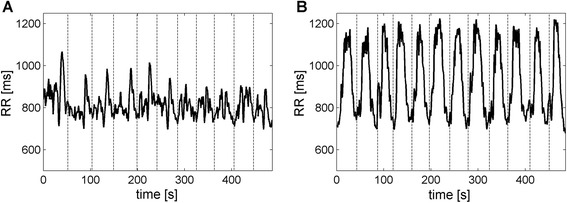
Examples of RR-interval time series during **a** EYT-A (*‘I Think The Saying’*) and **b** EYT-B (*‘Migraine B’*) obtained from one subject. In this example, EYT-A was repeated 11 times and EYT-B was repeated 12 times during 8 min. Each repetition of the sequence of movements is indicated by the dashed lines. During *‘I Think The Saying’* a lengthening of RR-intervals occurs at regular intervals whereas during *‘Migraine B’* a pronounced oscillation occurs in the RR-interval series

During EYT-A a prominent pattern was obvious in the RR-interval series, *i.e.*, a lengthening of RR-intervals occurred several times during the performance of the exercise (see Fig. 2A). This pattern was not reflected by SDNN. SDNN was lowest during CE2-A, whereas the other conditions showed a higher SDNN (EYT-A: 61 *vs.* 44 ms, p < 0.05; cf. Table 1) similar to Rest-A (67 ms), *i.e.* the overall variability was similar during these conditions.

In the frequency domain the VLF component (EYT-A: 6.68 ln ms^2^) was similar during the different conditions. The frequency of the peak oscillation in the VLF range was highest during CE1-A (0.028 Hz) and lowest during CE2-A (0.013 Hz, p < 0.05) whereas the frequency was intermediate during EYT-A (0.019 Hz) and during Rest (0.016 Hz). LF oscillations were largest during EYT-A (7.31 ln ms^2^) compared to all other conditions (CE1-A: 6.98 ln ms^2^; CE2-A: 6.52 ln ms^2^; Rest-A: 6.94 ln ms^2^; all comparisons: p < 0.05). The frequency of the peak oscillation in the LF range was 0.081 Hz and did not change during the different exercises. HF oscillations were larger during Rest-A (6.71 ln ms^2^) compared to CE1-A (5.44 ln ms^2^, p < 0.05) and CE2-A (5.04 ln ms^2^, p < 0.05). The frequency of the peak oscillations in the HF range were lowest during EYT-A (0.178 Hz) and highest during CE2-A (0.238 Hz, p < 0.05) whereas they were intermediate during CE1-A (0.208 Hz) and Rest-A (0.226 Hz).

### EYT-B exercise (‘Migraine B’)

The recorded videos showed that the sequence of body movements during the EYT-B exercise was repeated 13 times on average during 8 min (approx. 37 s per cycle corresponding to approx. 0.0275 Hz) whereas CE1-B was repeated 36 times on average (approx. 13 s per cycle corresponding to approx. 0.075 Hz). The average RR-interval during EYT-B was 774 ms. It was shorter during CE2-B (620 ms) and longer during Rest-B (878 ms) but only CE1-B (730 ms) and CE2-B differed significantly from Rest-B (p < 0.05). During EYT-B pronounced oscillations occurred in the RR-interval series (see Fig. 2B). The oscillations ranged from approx. 800 ms to 1100 ms. Accordingly, SDNN was largest during EYT-B whereas it was lowest during CE2-B (138 *vs.* 33 ms, p < 0.05), and intermediate during Rest-B (73 *vs.* 138 ms during EYT-B, p < 0.05).

The oscillations of the RR-interval series during EYT-B led to a substantial increase of the VLF component compared to Rest-B (9.32vs. 7.05 ln ms^2^, p < 0.05). The peak oscillation in the VLF range had a frequency of 0.026 Hz during EYT-B and was lower during Rest-B (0.012 Hz, p < 0.05) and during CE2-B (0.008 Hz, p < 0.05) whereas it was intermediate during CE1-B (0.018 Hz). The LF component was largest during CE1-B whereas it was lowest during CE2-B (8.98 *vs.* 5.47 ln ms^2^, p < 0.05). Here, the frequency of the peak oscillation was highest during Rest-B (0.090 Hz) and lowest during EYT-B (0.058 Hz, p < 0.05) whereas CE1-B and CE2-B were intermediate (0.070 Hz). The HF component did not show specific effects of EYT-B when compared to CE1-B or Rest-B but it decreased during CE2-B (6.68 *vs.* 3.82 ln ms^2^, p < 0.05). The frequency of the peak oscillation was highest during Rest-B (0.236 Hz) and lowest during EYT-B and CE1-B (0.185 Hz, p < 0.05).

## Discussion

In this study we found different effects of the two different EYT exercises on cardiac autonomic regulation. During the repetition of the sequence of movements of EYT-A (‘I Think The Saying’) pronounced LF oscillations occurred in the RR-interval series. The peak oscillation in this frequency range had frequency of 0.08 Hz which is four times the peak oscillation in the VLF band (0.02 Hz). In the VLF band the peak oscillation matched the repetition rate of the sequence of movements. Hence, the repetition of body movements had an impact on cardiac autonomic control. The oscillation in the LF band seemed to be induced by the different body positions and in particular by the different arm positions. The peak oscillation in the HF band was lowest during EYT-A indicating that the respiratory rate decreased during the exercise. Hence, EYT-A also seemed to have an impact on respiration. These effects were not observable during the control exercises. During EYT-B (‘Migraine B’) pronounced VLF oscillations appeared compared to Rest. The peak oscillation in the VLF band (0.028 Hz) again matched the repetition rate of the sequence of movements. During the control exercise with the same sequence of movements but without guided imagery the peak oscillation in the LF band matched the repetition rate of the control exercise (0.08 Hz). Hence, the repetition of standing, going to the squat and erecting again (as a continuous body movement) had a clear repetitive impact on cardiac autonomic control during EYT-B and its adapted control exercise. Similar to EYT-A the exercise slowed down the respiratory rate compared to the resting condition as indicated by the decrease of the peak oscillation in the HF. On the contrary, walking on the spot as another control exercise without an explicit repetition of specific body movements especially of the upper part of the body did not show any rhythmic impact on cardiac autonomic control.

How can the agreement of the repetition rate of the sequence of body movements with peak oscillations of HRV be explained? Generally, normal body movements such as actively standing up from the supine position clearly change the autonomic control, *i.e.* the average RR-interval decreases and HF component of HRV decreases [17]. Such movements may also contribute to VLF oscillations in long term Holter ECG recordings [18]. Other movements like *e.g.* squatting, raising or swinging the arms also lead to specific alterations of cardiovascular regulation and breathing [19–21]. These changes reflect reactions of the cardiac autonomic regulation to the particular movement. The present findings primarily reflect alterations caused by the repetition of the sequence of specific body movements of the EYT exercises.

In particular, the EYT-A exercise *‘I Think The Saying’* (and the control exercise CE1-A) comprised a sequence of different body postures which were strongly determined by the position of the arms. In all postures the arms were at least slightly raised and in most positions the arms show a shoulder flexion of 90° or more (see Fig. 1A, pictures 2 to 7). Such arm positions alter the tidal volume of respiration because they are accompanied by a decrease of inspiratory capacity and an increase of functional residual capacity [21]. Hence, also respiratory sinus arrhythmia, a contributing factor of HRV, is altered by the restrictions imposed on the tidal volume [22, 23]. The increased LF component during the performance of this EYT exercise had a peak oscillation at a frequency of 0.081 Hz which is four times the frequency of the repetitions of the sequence of body movements (0.02 Hz). Hence, each sequence of body movements (*i.e.* different arm positions) gave also rise to four oscillations in the beat-to-beat series. However, the association of the timing of these oscillations to the different body postures is difficult because a simultaneous recording of *e.g.* the position of the arms and the ECG was not available. Although the EYT-A exercise and CE1-A consisted of the same sequence of movements this effect was not visible during CE1-A. Taking into account that CE1-A was carried out faster than the EYT-A exercise, it seems that the lower speed of the EYT-A exercise altered cardiac autonomic regulation more differentiated than CE1-A. Furthermore, the EYT movements were performed with higher awareness and thus lower speed, an effect that also contributes to the described difference.

During EYT-B exercise *‘Migraine B’* (and the control exercise CE1-B) the pronounced oscillations of the RR-interval series were also caused by the repetition of the sequence of body movements of this exercise. An essential part of the body movements consists of slowly going to the squat and then standing up again (see Fig. 1B). During squat the RR-intervals and blood pressure increase immediately whereas this effect is reversed after standing up again [20, 24, 25]. Hence, the VLF oscillation of the RR-intervals during this EYT-B exercise reflects the repetition of the sequence of body movements (slowly going to the squat and erecting again) during the EYT-B exercise. The peak frequency of the oscillations in the VLF range (0.0265 Hz) was in agreement with the frequency of the repetition of the sequence of body movements. Hence, the aforementioned physiological changes during going to the squat and erecting were responsible for the synchronous effects of the body movements on cardiac autonomic regulation. For CE1-B the peak frequency of the oscillations in the LF range (0.07 Hz) again corresponded to the frequency of the repetition of the sequence of body movements. Hence, the oscillations were again evoked by the repetition of squatting and straightening up again. Notice that CE1-B was also carried out much faster than the EYT-B exercise (like in CE1-A, see above) because the awareness of the movements was much lower (no guided imagery).

What might be the physiological relevance of these findings? LF and VLF oscillations are associated with baroreflex sensitivity which is controlled by parasympathetic activity [26]. The enhancement of LF oscillations of the cardiovascular system (*e.g.* by means of slow breathing) improves baroreflex sensitivity and decreases blood pressure in patients with essential hypertension [27]. Hence, in patients with coronary heart disease, home-based Tai Chi training (3 times/week) together with conventional cardiac rehabilitation improved baroreflex sensitivity compared to conventional cardiac rehabilitation only [28]. In this study, the VLF and LF oscillations were induced by the repetition of specific body movements instead of *e.g.* slow respiration. Nevertheless, one may also expect an improvement of baroreflex sensitivity and decrease of blood pressure by the specific EYT exercises when performed *e.g.* three times per week for several weeks.

It has to be noted that guided imagery (without accompanying body movements) could also lead to alterations of the cardiac autonomic regulation. Different types of meditation (*e.g.* Chinese Chi and Kundalini Yoga) have a specific impact on HRV [29]. During Zen style meditation (calm sitting without accompanying imagery) breathing may be altered and even cardiorespiratory synchronization may occur [30]. Meditation clearly affects cardiac autonomic regulation because *e.g.* the LF component of HRV is often increased during meditation [29–32]. Furthermore, different emotional and mental states have an impact on autonomic control [33] and may even show distinct patterns of sympathetic and parasympathetic regulation [34]. However, in interventions which involve conscious body movements and guided imagery simultaneously (*e.g.* EYT or Tai Chi) the effects of guided imagery on HRV are masked by the large effects of the body movements.

A limitation of this study is that the order of exercises (EYT, CE1, CE2) was not randomized and the speed of performing CE1 was not controlled (the subjects were asked to perform all exercises with similar effort). In order to improve the comparability between the results of EYT and CE1 the performance speed should be controlled in further studies to obtain a similar amount of repetitions of body movements during EYT and CE1. On the other hand, the experience that the EYT exercises require a conscious attention and involvement which was obviously not present in the control activities reflects real life conditions. Another limitation is the lack of signals such as the movement status (*e.g.* tracking of arm position) and other physiological measurements (*e.g.* respiration). Such signals would help to better identify the interaction between body movements and alterations of cardiovascular regulation. In particular, *e.g.* the impact of different arm positions on HRV could be investigated in more detail using more sophisticated study settings. It also has to be noted that the body movements lead to augmentation of the VLF and LF component (see above). Particularly VLF oscillations may lead to nonstationarities of the beat-to-beat series and, hence, the spectral components contributing to the cardiac autonomic regulation may be overestimated [35].

## Conclusions

In summary, EYT exercises impose rhythmic effects on cardiac autonomic regulation through cyclic repetitions of a sequence of specific body movements, *i.e.* HRV shows oscillations simultaneously to the cyclic repetition of body movements. The effects on autonomic cardiac regulation depend on the specific sequence of body movements used during the exercise. Depending on the body movements each exercise has an impact on different parameters like *e.g.* thoracic pressure or venous return which are vital for cardiac autonomic control. We hypothesize that each EYT exercise leads to specific alterations of cardiac autonomic regulation if the exercise is practiced several times in a serial succession (in analogy to ‘normal’ exercise training which also leads to different adaptations which also affects cardiac autonomic regulation). Future studies should also focus on alterations of the autonomic control mediated by the specific emotional and mental states of each EYT exercise. Next steps will be to initiate longitudinal studies to monitor the effects of regular EYT practice, and thus the verification of specific exercises which might be used in a clinical setting.
